# Tickborne Relapsing Fever, Bitterroot Valley, Montana, USA

**DOI:** 10.3201/eid2102.141276

**Published:** 2015-02

**Authors:** Joshua Christensen, Robert J. Fischer, Brandi N. McCoy, Sandra J. Raffel, Tom G. Schwan

**Affiliations:** St. Patrick Hospital, Missoula, Montana, USA (J. Christensen);; University of Montana College of Health Professionals and Biomedical Sciences, Missoula (J. Christensen);; National Institute of Allergy and Infectious Diseases, Hamilton, Montana, USA (R.J. Fischer, B.N. McCoy, S.J. Raffel, T.G. Schwan)

**Keywords:** Borrelia hermsii, Ornithodoros hermsi, ticks, spirochetes, bacteria, tickborne relapsing fever, zoonoses, Bitterroot Valley, Montana, United States

## Abstract

In July 2013, a resident of the Bitterroot Valley in western Montana, USA, contracted tickborne relapsing fever caused by an infection with the spirochete *Borrelia hermsii*. The patient’s travel history and activities before onset of illness indicated a possible exposure on his residential property on the eastern side of the valley. An onsite investigation of the potential exposure site found the vector, *Ornithodoros hermsi* ticks, and 1 chipmunk infected with spirochetes, which on the basis of multilocus sequence typing were identical to the spirochete isolated from the patient. Field studies in other locations found additional serologic evidence and an infected tick that demonstrated a wider distribution of spirochetes circulating among the small mammal populations. Our study demonstrates that this area of Montana represents a previously unrecognized focus of relapsing fever and poses a risk for persons of acquiring this tickborne disease.

## Abstract 2

Persons in this region are at risk for acquiring this disease.

Seminal research on tickborne diseases of humans in North America began more than a century ago with the discovery in 1906 that an illness locally called black measles, which affected persons in the Bitterroot Valley of western Montana, USA, resulted from the bite of a bacteria-infected Rocky Mountain wood tick (*1**,**2*). What soon followed was the establishment of a multidisciplinary public health program to control this newly identified disease, now called Rocky Mountain spotted fever, which was caused by *Rickettsia rickettsii*, and a search was conducted for other diseases in nature that resulted from the bite of pathogen-infected ticks. These programs were based at a newly funded state laboratory in Hamilton in the Bitterroot Valley, a facility that was soon incorporated into the US Public Health Service and is now the Rocky Mountain Laboratories (RML) of the National Institute of Allergy and Infectious Diseases.

One of the many diseases studied at the RML since the early 1930s has been tickborne relapsing fever (RML, unpub. data) (*3**,**4*). In North America, this zoonosis is associated with 3 species of spirochetes, but most human cases are caused by *Borrelia hermsii*, which is found in scattered foci in the western United States and southern British Columbia, Canada (*5**,**6*). The specific vector of this spirochete is the *Ornithodoros hermsi* tick (*7*), which is found in higher-elevation coniferous forests where its preferred rodent hosts, primarily squirrels and chipmunks, are also found (*6*). In spite of the many decades of intensive research on ticks and tickborne diseases in the Bitterroot Valley, the tick *O. hermsi*, the spirochete *B. hermsii*, or an autochthonous human case of relapsing fever has not been observed in this region of Montana, until now. We report a case of tickborne relapsing fever in a person in this region.

## Case-Patient

The patient was a previously healthy 55-year-old man who sought care in July 2013 after a week of fevers. He had traveled widely over the previous months, including trips to Antarctica, New Zealand, Spain, Italy, and eastern Washington State, before returning to his home in the Bitterroot Valley of Montana. A week before his symptoms developed, he had moved part of a woodpile and noted rodent feces among the cut logs. He came to the emergency department of a local hospital because of fever, chills, night sweats, fatigue, nausea, vomiting, diarrhea, and malaise.

At initial evaluation, he had a temperature of 37.3°C; other vital signs were within reference ranges. Initial laboratory values included mildly increased levels of bilirubin, alkaline phosphatase, and aspartate aminotransferase, and mild transaminitis with an increased level of alanine aminotransferase (Table 1). A complete blood count showed thrombocytopenia (platelet count 51,000/mL). Hemoglobin level and leukocyte count were within reference ranges. He was given a presumptive diagnosis of an acute viral infection and discharged from the emergency department.

**Table 1:** Laboratory test results for serum from a 55-year-old man with tickborne relapsing fever on 2 dates compared with reference values, Montana, USA, 2013

Characteristic	Blood components examined*								
	Platelets/mL	Bilirubin, mg/dL	ALP, IU/L	AST, U/L	ALT, U/L	Serum lactate, mmol/L	PCT, μg/L	LDH, U/L	CK, U/L
Sample date									
Jul 20	51,000	1.7	234	89	97	ND	ND	ND	ND
Jul 22	29,000	6.8	414	215	144	3.9	7.99	495	602
Reference range or value	150–450,000	0.2–1.9	45–150	14–59	10–55	0.5–2.2	<0.05	300–600	20–200

*ALP, alkaline phosphatase; AST, aspartate aminotransferase; ALT, alanine aminotransferase; PCT, procalcitonin; LDH, lactate dehydrogenase; CK, creatine kinase; ND, not determined.

He returned 2 days later with confusion, tachycardia, hypoxemia, and a measurable fever (temperature 38.7°C). A physical examination identified a bilateral conjunctival suffusion, more notable in the right eye; cervical lymphadenopathy, tachypnea, tachycardia, splenomegaly, and major confusion. Additional laboratory tests showed a leukocyte count of 9,400 cells/mm^3^with 23% bands and a further reduced platelet count of 29,000/mL. Levels of bilirubin, alkaline phosphatase, aspartate aminotransferase, alanine aminotransferase, serum lactate with lactic acidosis, procalcitonin, and total creatine kinase were all highly increased above reference values (Table 1). Urinalysis showed a 2+ protein level, and a chest radiograph showed diffuse bilateral pulmonary infiltrates.

He was admitted to the hospital and given intravenous ceftriaxone and oral doxycycline pending further examination. A peripheral smear was prepared, which showed thrombocytopenia and multiple extracellular spirochetes. A coded diagnostic specimen of the patient’s whole blood was sent to the RML for identification of the spirochetes. His hospital course was notable for acute respiratory failure caused by acute respiratory distress syndrome, metabolic encephalopathy, and uveitis of the right eye. His sepsis and acute respiratory distress syndrome resolved, and his condition rapidly improved after treatment with ceftriaxone and doxycycline. On hospital day 5, he was discharged and given a 14-day course of oral doxycycline.

He was seen as an outpatient and showed resolution of abnormalities and symptoms, except for decreased visual acuity of the right eye that ultimately required a corrective lens. Results of serologic tests performed at a commercial diagnostic laboratory were negative for antibodies against *Coxiella burnetii*, *Brucella* spp., *Francisella tularensis*, *Leptospira interrogans*, *Treponema pallidum*, hantavirus (Sin Nombre virus), Colorado tick fever virus, cytomegalovirus, Epstein-Barr virus, and hepatitis A, B, and C viruses. An IgM test result for Lyme disease was equivocal but was probably cross-reactive because of the *B. hermsii* infection. A convalescent-phase serum sample obtained 6 weeks after onset of symptoms was sent to the Centers for Disease Control and Prevention (Fort Collins, CO, USA). This sample was positive for *B. hermsii* by enzyme immunoassay and Western blotting.

## Materials and Methods

The coded diagnostic sample of the patient’s blood obtained on July 22, 2013 was received at RML 3 days later. Thin blood smears were prepared on microscope slides, fixed with 100% methanol, and examined by using indirect fluorescent antibody staining with monoclonal antibodies H9724 (*8*) and H9826 (*9*) to identify spirochetes.

Motile spirochetes were observed in the blood by dark-field microscopy, and 100 µL of sample was inoculated intraperitoneally into 1 laboratory mouse (*Mus musculus*) to amplify and isolate spirochetes as described (*10*). Spirochetes in mouse blood were isolated in Barbour-Stoenner-Kelly x medium (*11**,**12*) containing 12% rabbit serum.

Multilocus sequence typing (MLST) of spirochetes was performed with purified genomic DNA samples extracted from bacterial cultures. Seven genetic loci were examined: partial sequences for the 16S rRNA (1,273 bp) and intergenic spacer (663 bp); and complete sequences for *flaB* (1,002 bp), *gyrB* (1,902 bp), *glpQ* (1,020 bp), *fhbA* (555 bp), and *vtp* (627 bp). Approximately 7,042–7,111 bp were determined for each isolate on which to base species identifications. The primers, PCR conditions, and methods for DNA sequencing have been described (*13**–**15*).

Given the patient’s date of onset of illness, travel history, and reported incubation periods for the infection (average 7 days, range 4–18 days) (*16*), we concluded that he was possibly infected on his own property. Therefore, we conducted a short on-site investigation to search for supporting evidence that he could have been infected there. Live traps (Tomahawk Live Traps, Hazelhurst, WI, USA, and H.B. Sherman Live Traps Inc., Tallahassee, FL, USA) were set on the property on the evening of August 6 to the morning of August 8, 2013. The animals captured were anesthetized with isoflurane, blood was collected from the tail vein, standard morphometric data were collected for each animal, and they were identified to species on the basis of published descriptions of Montana wildlife (*17*). A skin punch biopsy was taken from 1 external ear for DNA extraction and determination of the mitochondrial *cytB* gene sequence to confirm the identifications (*18*), and the animals were released at the location of capture.

This work was conducted with approval of the RML Animal Care and Use Committee (protocol #2012–029 and #2013–035), the Montana Department of Fish, Wildlife and Parks (permit #2013–104) and the Bitterroot National Forest (permit #BIT201310). Work with animals was conducted in accordance with the institution’s guidelines for animal husbandry, and followed the guidelines and basic principles in the Public Health Service Policy on Humane Care and Use of Laboratory Animals.

Blood samples from the wild rodents were examined for spirochetes as wet mounts with a dark-field microscope, and as fixed, Giemsa-stained thin blood smears with a bright-field microscope. Serum samples (1:100 dilution) were tested by immunoblot with a *B. hermsii* whole-cell lysate and purified recombinant glycerophosphodiester phosphodiesterase (rGlpQ) as described (*19**,**20*).

CO_2_ traps baited with dry ice were set at 2 locations on the property, including the woodpile that the patient had begun to move 6 days before becoming ill, to attract ticks. We also collected debris from the woodpile and processed the material by using Berlese funnels in an attempt to extract live ticks.

## Results

**Figure 1:**
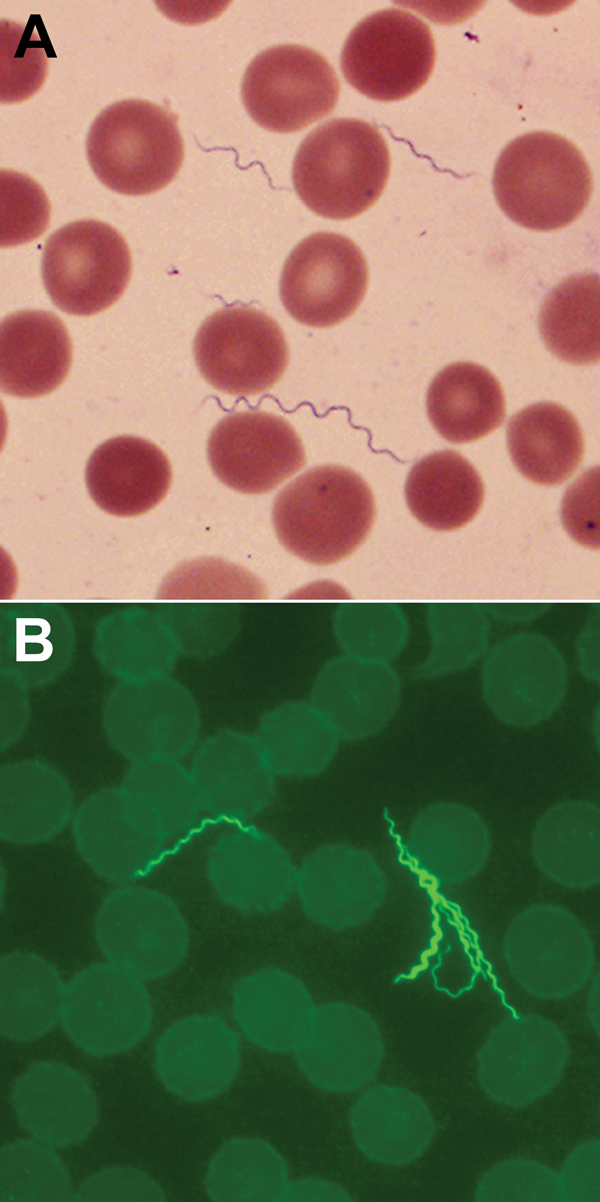
A) Spirochetes in blood smear of a 55-year-old man with tickborne relapsing fever, Bitterroot Valley, Montana, USA (Giemsa stain). Erythrocyte diameters are ≈6–8 µm. B) Spirochetes in blood smear of the patient visualized by indirect immunofluorescent antibody staining with mouse monoclonal antibody H9724 and goat anti-mouse antibody conjugated with fluorescein isothiocyanate (original magnification ×1,000).

Spirochetes in thin blood smears for the patient were visualized by staining with Giemsa and were positive by indirect fluorescent antibody staining with monoclonal antibodies H9724 and H9826, which identified the bacterium as *B. hermsii* (Figure 1). The spirochetes grew through 2 passages in mice and were isolated in the second passage in vitro culture. Stocks of the spirochete culture were frozen at –80°C, and the isolate was designated *B. hermsii* COR. MLST identified the isolate as *B. hermsii* that belonged to genomic group I (GGI) (*13*,*21*).

The patient lived on a rural, 40,469 m^2^ area located east of Corvallis in Ravalli County, Montana, ≈12 km northeast of RML on the eastern slope of the Bitterroot Valley at an elevation of ≈1,250 m. The site included his house and a nearly pure stand of secondary growth yellow pine (*Pinus ponderosa*) and several large rock outcrops. We examined the site, which included cut yellow pine cones, feces, and shredded bark in the woodpile, and found evidence of rodent activity.

**Figure 2:**
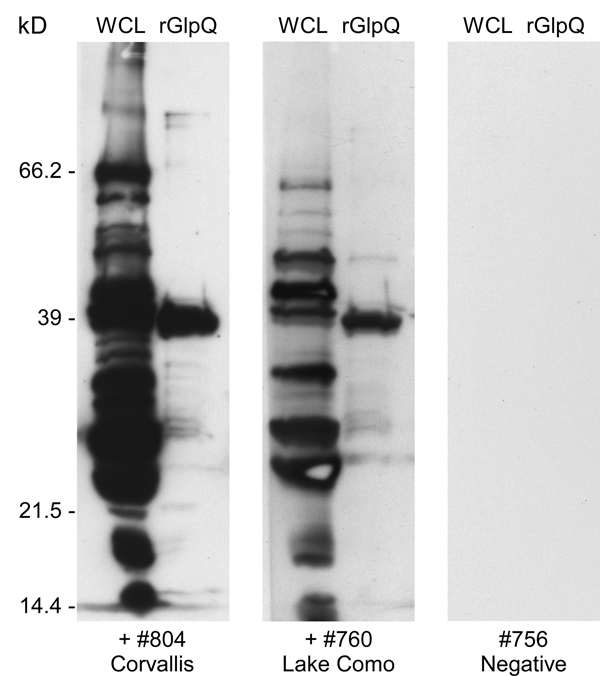
Immunoblot analysis of serum samples from 2 animals for *Borrelia hermsi*, Bitterroot Valley, Montana, USA. Samples were tested with *B. hermsii* whole cell lysates (WCL) (left lanes) and purified recombinant glycerophosphodiester phosphodiesterase (rGlpQ) (right lanes). +#804 Corvallis, seropositive sample from yellow-pine chipmunk (*Tamias amoenus*) trapped on patient’s property; +#760 Lake Como, seropositive sample from red-tailed chipmunk (*T. ruficaudus*) trapped at Lake Como, Montana; #756 Negative, seronegative sample from chipmunk.

A total of 150 trap-nights captured 2 deer mice (*Peromyscus maniculatus*) and 7 yellow-pine chipmunks (*Tamias amoenus*), 1 of which was spirochetemic at the time of capture. Spirochetes in fixed thin blood smears were reactive with monoclonal antibodies H9724 and H9826, which also identified these bacteria as *B. hermsii*. Spirochetes in the chipmunk’s blood were passed through 4 laboratory mice and isolated in the second passage by using in vitro culture. Stocks of this culture were also frozen at −80°C and the isolate was designated *B. hermsii* COC-807. In addition, a serum sample from 1 of the 6 remaining chipmunks was seropositive by immunoblot analysis with antibodies binding to numerous spirochete proteins and purified rGlpQ (Figure 2). MLST sequences for the same 7 loci for the spirochete isolated from the chipmunk were identical to sequences determined for the spirochete isolated from the patient.

**Figure 3:**
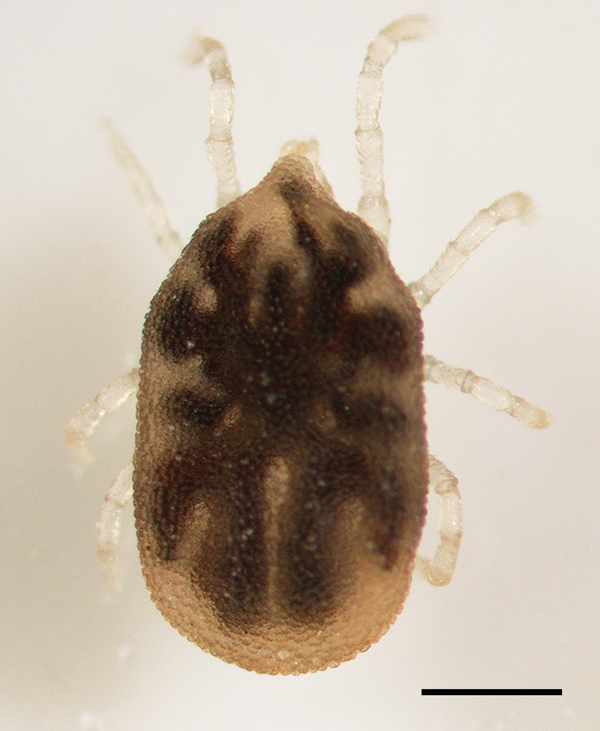
*Ornithodoros hermsi* nymph collected from the property of a 55-year-old man with tickborne relapsing fever, Bitterroot Valley, Montana, USA. Scale bar = 0.5 mm.

We collected debris from the woodpile for processing by using Berlese funnels, and on the evening of August 6, 2013, we placed a CO_2_ trap baited with a block of dry ice among the logs. The following morning, we found 3 *O. hermsi* nymphs in the substrate under the trap (Figure 3), and during the following weeks we found 6 more live ticks (4 nymphs, 2 males) from the debris that we processed by using the Berlese funnels. We fed 6 ticks individually on mice and triturated the remaining 3 ticks. The material from these 3 ticks was suspended in phosphate-buffered saline and injected into mice.

From the blood of these experimentally exposed mice, we isolated spirochetes that originated from 4 (2 nymphs, 2 males) of the 9 ticks. These isolates were established in culture, preserved as frozen stocks, and designated COT-5, COT-6, COT-7, and COT-8. MLST of the 7 loci identified COT-5 and COT-8 as *B. hermsii* belonging to GGI and identical to isolates from the patient and chipmunk; COT-7 belonged to GGII (7,111-bp sequence determined for this isolate). Isolate COT-6 was a mixed infection that demonstrated that 1 tick was infected with 2 *B. hermsii* strains that represented both genomic groups.

Once we identified the specific tick vector, we covered the woodpile with plastic tarpaulins and fumigated it with an aerosol from 2 cans of an over-the-counter insect fogger. This fogger contained 0.10% tetramethrin and 0.60% cypermethrin as the active ingredients.

At the time of the patient’s illness, we had already begun a preliminary field study in the Bitterroot National Forest adjacent to the Bitterroot Valley to search for evidence that *B. hermsii* might be present. We investigated 2 sites, Lake Como (elevation 1,450 m) and Hughes Creek (elevation 1,575 m), which are ≈24 km and 72 km south-southwest of RML, respectively. At these 2 locations, we captured 8 species of rodents that included 178 animals (Table 2). None of the animals exhibited microscopically detectable spirochetes in their blood when captured. However, immunoblot analysis of serum samples demonstrated that 9 animals representing 4 species were seropositive, which indicated they had been previously infected with spirochetes. These animals included 1 red-tailed chipmunk (*Tamias ruficaudus*) at Lake Como (Figure 2) and 5 chipmunks of the same species at Hughes Creek. Additional seropositive animals at Hughes Creek included 1 northern flying squirrel (*Glaucomys sabrinus*), 1 golden-mantled ground squirrel (*Callospermophilus lateralis*), and 1 western jumping mouse (*Zapus princeps*).

**Table 2:** Animals serologically analyzed for infection with *Borrelia hermsi* in 3 locations, Bitterroot Valley, Montana, USA, July 8–September 13, 2013*

Species captured	Common name	No. captured by region			
		Corvallis	Hughes Creek	Lake Como	Total
*Urocitellus columbianis*	Columbian ground squirrel	0	53	0	53
*Callospermophilus lateralis*	Golden-mantled ground squirrel	0	2†	35	37
*Tamias ruficaudus*	Red-tailed chipmunk	0	28‡	9†	37
*Tamias amoenus*	Yellow-pine chipmunk	7†	1	0	8
*Tamiasciurus hudsonicus*	American red squirrel	0	5	0	5
*Glaucomys sabrinus*	Northern flying squirrel	0	1†	0	1
*Peromyscus maniculatus*	Deer mouse	2	37	2	41
*Zapus princeps*	Western jumping mouse	0	5†	0	5
Total	NA	9	132	46	187

*NA, not applicable. †One animal was seropositive. ‡Five animals were seropositive.

On September 12, 2013, we collected soil litter from under a tick trap baited with dry ice at Hughes Creek, from which we extracted 1 *O. hermsi* nymph. This tick transmitted spirochetes when it fed on a laboratory mouse. We isolated spirochetes from the infected blood and designated this isolate HCT-4. MLST identified the spirochete as *B. hermsii* that belonged to GGI, and the spirochete was identical to the other GGI isolates described above. Sequences for the 16S rRNA, *flaB*, *gyrB*, *glpQ*, *vtp*, *fhbA*, and intergenic spacer loci for *B. hermsii* isolates COR, COC-807, COT-7, and HCT-4 have been deposited in GenBank under accession nos. KJ995774–KJ995801.

## Discussion

This study reports an autochthonous human case of tickborne relapsing fever caused by *B. hermsii* in the Bitterroot Valley of western Montana. The patient had many of the typical clinical signs and symptoms of this infection (*22*), with the notable exception of a unilateral uveitis that resulted in permanent damage to the right eye for which a corrective lens was required. Various forms of uveitis have been described with other spirochetal diseases, including Lyme disease (*23*), syphilis (*24*), and leptospirosis (*25*). Ocular complications, including uveitis, have been reported for cases of relapsing fever caused by other species of spirochetes (*16*), such as in troops in Libya during World War II (*26*). Iritis (anterior uveitis) has been associated with a few cases of relapsing fever in the southwestern United States (*27*), which were probably caused by *B. turicatae*. However, specific ocular involvement resulting from an infection with *B. hermsii* is rare.

Horton and Blaser (*28*) described endophthalmitis in a 3-month-old infant who contracted relapsing fever in a mountain cabin in Colorado. Although no spirochetes were isolated or identified, the ecologic and geographic setting suggests that the patient was infected with *B. hermsii*. The only case of uveitis purported to be caused by *B. hermsii* was in a 12-year-old boy exposed in eastern Oregon (*29*). Although no spirochetes were observed or identified in this patient, the clinical course and cross-reactive serologic test result for *B. burgdorferi*, a cause of Lyme disease, led the authors to conclude that the patient had been infected with *B. hermsii*. From the patient with uveitis in our study, we isolated the spirochete and confirmed its identity as *B. hermsii* by performing extensive molecular characterization. We are unaware that this etiologic confirmation was made for any case of relapsing fever with uveitis, regardless of the species of spirochete involved.

Our conclusion that the patient was infected locally is supported by his restricted travel just before onset of illness, our findings of infected ticks and an infected chipmunk on his property, and extensive DNA sequence analysis that demonstrated that the isolates of *B. hermsii* from patient, chipmunk, and 2 of the ticks were identical. These results and our findings of seropositive animals and an infected *O. hermsi* tick south of the study site showed that the slopes of the Bitterroot Valley and surrounding areas represent a newly identified area to which *B. hermsii* spirochetes are endemic, which has the potential for being a source of human infections in this region of Montana.

Before our investigation, all known human cases of tick-borne relapsing fever caused by *B. hermsii* in Montana had originated on Wild Horse Island in Flathead Lake, in the northwestern part of the state. The first documented outbreak occurred there during the summer of 2002 (*10**,**30*). Five persons, all of whom resided elsewhere, became infected while sleeping in a tick-infested cabin during a family reunion. In 2004, three more persons became infected with *B. hermsii* while sleeping in another recreational cabin on the island, a short distance east of where the first outbreak occurred (*21*). Isolates of *B. hermsii* were obtained from the 2 patients infected in 2002 (*10*) and the 3 patients infected in 2004 (*21*). MLST of the isolates from these 5 patients showed that both genomic groups of *B. hermsii* were present on the island and in the same cabin (*21*).

We recently showed experimentally that 1 *O. hermsi* tick can become superinfected with spirochetes that belonged to both genomic groups and later transmit both types of bacteria simultaneously during a subsequent feeding (*31*). During our onsite investigation of the patient’s property, we found 1 *O. hermsi* tick that was naturally infected with both genomic groups of spirochetes, which were co-transmitted during a single feeding on a mouse.

The patient with relapsing fever described in this report represents another example of an atypical exposure by becoming infected during a daytime activity. Although *O. hermsi* ticks are nocturnal and typically feed at night, persons who disturb materials infested with these ticks during the day might be bitten and become infected. We investigated a similar daytime exposure for a relapsing fever patient who was bitten by ticks while moving rodent-contaminated debris at Mount Wilson Observatory in Los Angeles County, California, USA (*20*). However, most persons in whom relapsing fever caused by *B. hermsii* develops are exposed at night in recreational cabins that are not the patient’s primary residence.

Among wild rodents we sampled, 7 (70%) of 10 animals that were seropositive were chipmunks, and 1 yellow-pine chipmunk was infected with *B. hermsii* when captured. In other areas of the western United States, these animals play a major role as hosts for *O. hermsi* ticks and *B. hermsii* (*32**–**35*). Therefore, our observations extend considerably the geographic range for chipmunks involved in a natural enzootic focus of relapsing fever.

Residents and visitors to the Bitterroot Valley need to be alerted that there is the potential for becoming infected locally with the relapsing fever spirochete *B. hermsii*. Tickborne relapsing fever should be considered when patients seek treatment for a history of recurrent, acute febrile episodes. Confirmation of the infection is made most often by visualizing spirochetes in a stained, thin blood smear (Figure 1) (*36*) made during a febrile episode and examined by a trained medical technologist, as was performed for the patient in our study. In addition, this area of Montana has long been a popular tourist destination for visitors from other regions of the United States, where the opportunity exists to enjoy many outdoor recreational activities. Health care providers in other parts of the country need to be aware that persons spending time outdoors in and around the Bitterroot Valley of Montana may be exposed to spirochetes causing relapsing fever in this newly identified disease-endemic area far from their place of residence.
